# Supplementary material for: Ni-Catalyzed Enantioselective Intramolecular Mizoroki–Heck Reaction for the Synthesis of Phenanthridinone Derivatives
Source: J Org Chem. 2023 Jun 15;88(13):8203–26. doi: 10.1021/acs.joc.3c00202 (PMC10337041; doi:10.1021/acs.joc.3c00202)
Supplement: Supplementary file 2 — jo3c00202_si_002.zip [file jo3c00202_si_002.zip › Amides (N-MOM)/1e-I iPr-NMOM-ArI/13C iPr-NMOM-ArI 1e-I/7/pdata/1/email_DR-112-082 F14-28 DMSO heat C13_7_1.pdf]

DR-112-082 F14-28 DMSO heat C13
C13APT DMSO {D:\nmrusers\malachowski} BM 16

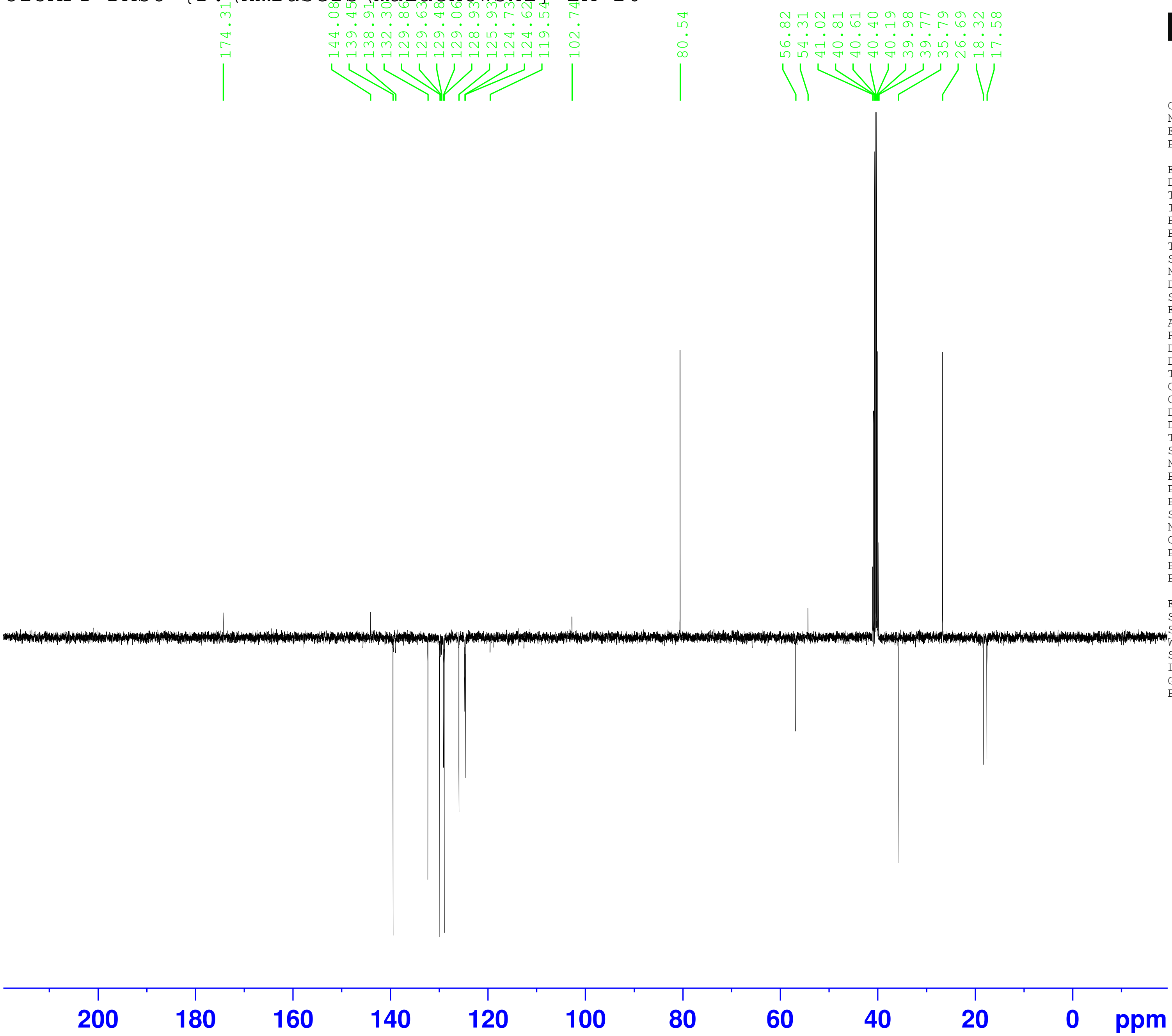


Current Data Parameters

| | |
|---|---|
| NAME | DR-112-082 F14-28 DMSO heat C13 |
| EXPNO | 7 |
| PROCNO | 1 |

F2 - Acquisition Parameters

| | | |
|---|---|---|
| Date_ | 20211022 | |
| Time | 23.22 | h |
| INSTRUM | spect | |
| PROBHD | Z104450_0352 ( | |
| PULPROG | jmod | |
| TD | 65536 | |
| SOLVENT | DMSO | |
| NS | 3024 | |
| DS | 4 | |
| SWH | 24038.461 | Hz |
| FIDRES | 0.733596 | Hz |
| AQ | 1.3631488 | sec |
| RG | 203 | |
| DW | 20.800 | usec |
| DE | 6.50 | usec |
| TE | 350.0 | K |
| CNST2 | 145.0000000 | |
| CNST11 | 1.0000000 | |
| D1 | 2.00000000 | sec |
| D20 | 0.00689655 | sec |
| TD0 | 1 | |
| SFO1 | 100.6278593 | MHz |
| NUC1 | 13C | |
| P1 | 8.00 | usec |
| P2 | 16.00 | usec |
| PLW1 | 56.13299942 | W |
| SFO2 | 400.1516006 | MHz |
| NUC2 | 1H | |
| CPDPRG[2 | waltz65 | |
| PCPD2 | 90.00 | usec |
| PLW2 | 12.14200020 | W |
| PLW12 | 0.24368000 | W |

F2 - Processing parameters

| | | |
|---|---|---|
| SI | 32768 | |
| SF | 100.6177975 | MHz |
| WDW | EM | |
| SSB | 0 | |
| LB | 1.00 | Hz |
| GB | 0 | |
| PC | 1.40 | |
